# Investigating the role of tumour-to-skin proximity in predicting nodal metastasis in breast cancer

**DOI:** 10.1007/s10549-023-07230-5

**Published:** 2024-02-03

**Authors:** Thiviya Sivakanthan, J. Tanner, B. Mahata, A. Agrawal

**Affiliations:** 1 University of Cambridge, Cambridge, UK; 2 Cambridge University Hospitals, Cambridge, CB2 0QQ UK

**Keywords:** Tumour to skin, Nodes, Metastasis, Sentinel, Axilla, Breast cancer

## Abstract

**Background:**

Understanding the factors influencing nodal status in breast cancer is vital for axillary staging, therapy, and patient survival. The nodal stage remains a crucial factor in prognostication indices. This study investigates the relationship between tumour-to-skin distance (in T1–T3 tumours where the skin is not clinically involved) and the risk of nodal metastasis.

**Methods:**

We retrospectively reviewed data from 100 patients who underwent neoadjuvant chemotherapy (NACT). Besides patient demographics and tumour variables, a radiologist retrospectively reviewed pre-operative MRI to measure tumour-to-skin distance. R core packages were used for univariate (χ2 and T-Wilcoxon tests) and bivariate logistic regression statistical analysis.

**Results:**

Of 95 analysable datasets, patients’ median age was 51 years (IQR: 42–61), 97% were symptomatic (rest screen detected), and the median tumour size was 43 mm (IQR, 26–52). On multivariate analysis, increasing invasive tumour size (*p* = 0.02), ER positivity (*p* = 0.007) and shorter tumour-to-skin distance (*p* = 0.05) correlated with nodal metastasis.  HER2 was not included in multivariate analysis as there was no association with nodal status on univariate analysis. In node-positive tumours, as tumour size increased, the tumour-to-skin distance decreased (*r* = − 0.34, *p* = 0.026). In node-negative tumours, there was no correlation (*r* = + 0.18, *p* = 0.23).

**Conclusion:**

This study shows that non-locally advanced cancers closer to the skin (and consequent proximity to subdermal lymphatics) are associated with a greater risk of nodal metastasis. Pre-operative identification of those more likely to be node positive may suggest the need for a second-look USS since a higher nodal stage may lead to a change in therapeutic strategies, such as upfront systemic therapy, node marking, and axillary clearance without the need to return to theatre following sentinel node biopsy.

## Background

An ultrasound scan (USS) and clinical examination are the current standard pre-operative assessment of axillary nodal status at diagnosis of new breast cancer. If these are negative, sentinel lymph node biopsy (SLNB) is performed. Those that appear abnormal undergo core needle biopsy or fine needle aspirate (FNA) and, if positive, undergo axillary node clearance (ANC). Node-positive cancers (with consequent higher N-Nodal stage in TNM staging for cancers) are associated with a poorer prognosis [1].

Larger breast tumours (higher T-Tumour stage in TNM) are associated with a higher rate of node positivity compared to smaller cancers [2–4]. However, smaller tumours can metastasise to lymph nodes, suggesting factors aside from tumour size are associated with node metastasis. Lymphovascular invasion (LVI) [5] is a common tumour feature influencing nodal metastasis. The immune profile of the node may also be altered by the tumour cells, allowing them to metastasise to the node by creating an immunosuppressive pre-metastatic niche [6]. Tumour cells can induce steroid biosynthesis in T cells, allowing the tumour cells to evade immunity and contribute to metastatic potential [7].

Studies have investigated the relationship between tumour-to-skin distance (TSD) and lymph node metastasis. A systematic review suggests that smaller TSD and tumours with associated architectural distortion on USS were important predictors of axillary node metastasis [8]. Within this review, a study by Bae et al. [5] investigated the effects of different USS features on nodal involvement and found that smaller TSD was associated with a higher nodal metastasis rate. By only including T1–3 stage breast cancers, the study ensured this association was independent of any clinical skin involvement seen in T4 breast cancer. Two retrospective studies in 2006 and 2015 found that smaller TSD was associated with increased node metastasis risk [9, 10]. Both studies excluded T4-stage breast cancer. One proposed theory for this association is that the lymphatic supply of the breast is less abundant in the parenchyma than in the superficial dermal and subdermal layers, allowing breast cancers closer to the skin to metastasise more easily, regardless of size [11, 12]. This is supported by studies that show LVI is a statistically significant factor associated with axillary node metastasis even in T1–2 stage breast cancer [5, 13]. However, whilst studies have investigated the relationship between LVI and TSD to axillary lymph node metastasis independently, no studies definitively show an association between TSD and LVI.

The aims of this study are:

- To investigate whether tumours close to the skin had higher subsequent sentinel node positivity in those with previously node-negative early breast cancer
- To understand what radiological (tumour size and TSD) and biological features will help identify patients who may benefit from a second-look USS and core biopsy preoperatively, allowing them to avoid repeat axillary surgery and delay in adjuvant therapy.

## Methods

### Study design

After internal institutional approval, retrospective data were collected from the electronic patient records of 100 breast cancer patients who underwent axillary surgery after neoadjuvant chemotherapy (NACT) and had pre-operative breast MRIs. The main reason for selecting this cohort was because performing a baseline MRI scan in patients recommended NACT is routine in our centre. Most have follow-up MRIs for assessment of response mid-chemotherapy, although an Ultrasound-only assessment may replace them. If the breast appears conservable and is patient preference, then an end-of-chemotherapy MRI is used to measure the final imaging response (in comparison with previous imaging) as well as to assist surgical planning. Hence, this will have been a cohort with the most complete MRI dataset to ascertain TSD on static operator-independent images.

Different tumour characteristics, including tumour receptor (ER, PR, and HER2) status, LVI status, invasive grade, tumour size, and TSD, were analysed with respect to nodal status.

- Select patients who had MRI scans (to gain the most consistent TSD measurements for retrospective data)
  i. Inclusion criteria:
    Patients who had treatment between August 2015 and August 2020.
    Female patients over 18 with breast cancer.
    Either node negative (underwent sentinel node biopsy) or node positive (underwent axillary node clearance); however, our practice has evolved to Targeted Axillary Dissection (after node clipping pre-NACT).
  ii. Exclusion criteria: T4 cancers and patients with no MRI scans.
  iii. All patients had also received NACT. The MRI scans used were before NACT.
  iv. Analyse retrospectively 50 upfront node-positive cancers and 50 node-negative cancers, some of which were positive on sentinel node biopsy.
    b. Measure TSD using MRI scans. We used the closest TSD on MRI. For multifocal cancers, we used the area of invasive disease closest to the skin as the target for measurement.
    c. Compare TSD between those who were found to be node-positive vs. node-negative
    d. Use retrospective data to identify what radiological (tumour size and TSD) and biological factors could be used to identify those at risk of nodal metastasis.
    e. Biological factors such as receptor status and tumour grade were assessed using pre-NACT core biopsy histology.
    f. Identify if those factors deemed significant were related to TSD.

### Statistical analysis

R core packages were used for statistical analysis (R Core Team (2022)). Pearson test *χ*^2^ (for qualitative variables) and T-Wilcoxon test for paired samples (for quantitative variables) were applied to compare individual tumour characteristics between node-positive and node-negative tumour groups [9, 14]. Logistic regression analysis investigated the relationship between TSD and other tumour variables significant on univariate analysis. Further, bivariate logistic regression analysis was performed to identify which of those variables significant on univariate analysis was still significant when controlling for other clinicopathologic variables [5].

## Results

Of 95 analysable datasets, the median age of the patients was 51 years (IQR, 42–61). Five patients were excluded due to the inaccessibility of the MRI scans. 97% were symptomatic, and the rest were detected on 3-yearly UK NHS breast screening between age 50 and 70. The median tumour size was 43 mm (IQR, 26–52). The majority were non-specific tumour (NST) type (n-88); the rest were ILC (n-4) and others (n-3). All node-positive patients on USS-guided biopsy underwent ANC (n-36). All clinically node-negative patients underwent SLNB (n-59); of SLNBs, 11 were node-positive (of which 10 underwent ANC and one axillary radiotherapy).

All 47 node-positive patients received NACT for TNBC (n-3), ER-HER2 + (n-0), ER + HER2 + (n-17), and ER + HER2-(n-27). All 48 node-negative patients received NACT for TNBC (n-22), ER-HER2 + (n-8), ER + HER2 + (n-9), and ER + HER2-(n-9).

Table 1 shows the increasing ratio between node-positive and node-negative status with increasing tumour stages.

**Table 1:** Tumour and nodal stage

Tumour stage	T1	T2	T3	Missing	Total
Node positive	1	19	23	4	47
Node negative	11	29	7	1	48
Total	12	48	30	5	95

T1–3, according to TNM classification

**Fig. 1:**
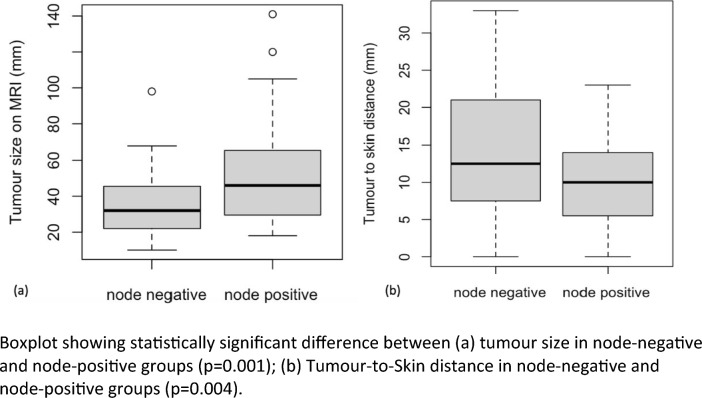
Association of tumour size and TSD with nodal status. Boxplot showing statistically significant difference between **a)** tumour size in node-negative and node-positive groups (*p* = 0.001) and **b)** TSD in node-negative and node-positive groups (*p* = 0.004)

**Fig. 2:**
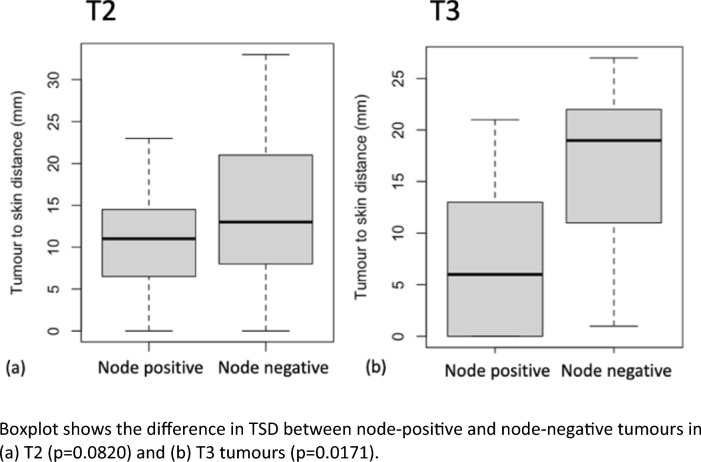
Association of TSD with nodal status. Boxplot shows the difference in TSD between node-positive and node-negative tumours in **a)** T2 (*p* = 0.0820) and **b)** T3 tumours (*p* = 0.0171)

Fig. 1 shows significantly higher node positivity (T test) with increasing tumour size (*p* = 0.001) and decreasing TSD on MRI (*p* = 0.004). The median TSD in the node-positive group was 10 (IQR, 5.5–10) mm. The median TSD for the node-negative group was 12.5 (IQR, 7.75–21) mm. Figure 2 shows the difference in TSD between node-positive and node-negative tumours in (a) T2 (*p* = 0.0820) and (b) T3 tumours (*p* = 0.0171).

The median TSD of the 11 patients who were node-negative on pre-operative USS but found to be node-positive after SLNB was 8 mm (IQR, 4.5–14.5 mm). Of these 11 patients, 1 was T1, 5 were T2, and 5 were T3.

*χ*^2^ tests showed that the TSD was insignificant (*p* = 0.082) in T2 tumours (55% of the cohort) and significant (*p* = 0.0171) in T3 tumours (33%). The T1 subgroup was too small for meaningful analysis.

Further, of the 15 tumours less than 3 mm from the skin, 10 were node-positive (66.67%). Of the 80 tumours over 3 mm from the skin, 37 (46.25%) were node-positive (*p* = 0.147). 3 mm was used as the superficial lymphatic drainage system has been identified to be within 3 mm from the skin [15].

Table 2 shows that node-positive tumours were more likely to have a positive LVI status (*p* = 0.002), positive ER status (*p* < 0.001), and positive PR status (*p* = 0.003). HER2 status was not associated with nodal status (*p* = 0.939).

**Table 2:** Association of tumour biology with nodal status

	Node positive	Node negative	*χ*^2^ value	*P* value
ER + ve	44	20	29.15	< 0.001
ER−ve	3	28		
PR + ve	18	31	4.53	0.03
PR−ve	2*	17		
LVI + ve	15	3	9.31	0.002
LVI−ve	32	42		
HER2 + ve	17	17	< 0.0059	0.939
HER2−ve	30	31		

*ER* Oestrogen Receptor, *PR* Progesterone Receptor, *LVI* Lymphovascular invasion, *HER2* Human epidermal growth factor-2

*PR status missing for 27 patients

### Relationship between significant tumour characteristics and TSD

Linear correlation analysis found that in node-positive tumours, as tumour size increased, the TSD decreased (*r* = − 0.340, *p* = 0.026). In node-negative tumours, there was no such correlation between size and TSD (*r* = + 0.18, *p* = 0.23).

Table 3 shows that on multivariate analysis, node positivity was associated with ER-positive tumours (*p* = 0.007) and tumours with a larger invasive size (*p* = 0.02). A shorter TSD was associated with increased node positivity with a borderline significance (*p* = 0.05).

**Table 3:** Correlation of tumour factors with nodal metastasis

Tumour characteristic	Multivariate analysis (p-value)	Correlation with nodal positivity
Larger tumour size	0.10	No correlation
Increasing invasive tumour size	0.02	+ ve
ER + ve	0.007	+ ve
HER2 + ve	Not included	No correlation on univariate
LVI + ve (18/95)	0.5	No correlation
Shorter TSD	0.05	+ ve

*ER* Oestrogen Receptor, *PR* Progesterone Receptor, *LVI* Lymphovascular invasion, *HER2* Human epidermal growth factor-2

## Discussion

Analysis of the whole cohort suggested an association of smaller TSD with positive nodal status (*p* = 0.004). This is in keeping with other studies [5, 8–10, 12]. Bae et al. indicated that a smaller TSD (*p* = 0.04) and associated architectural distortion (*p* = 0.003) were independent predictive factors for axillary lymph node metastasis in T1–2-staged breast cancers. In the study by Ojha et al., 88.7% of tumours with a TSD < 3 mm were node-positive and only 47% were positive in those with a TSD > 3 mm. Eom et al. reported that TSD < 3 mm was associated with more axillary nodal metastasis (*p* = 0.039) [10]. Our study found a greater proportion of tumours < 3 mm from the skin to be node-positive (66.67%) compared to tumours > 3 mm from the skin (46.25%), although this was not statistically significant. This may be due to the limited sample size; only 15 tumours were found < 3 mm from the skin. Cunningham et al. found that the threshold at which tumour-to-skin proximity becomes significant in T1–T2 cancers is 14 mm. Essa et al. group reported a similar distance (15 mm) [16].

On multivariate analysis, our data showed TSD to be of borderline significance (*p* = 0.05). Unlike the studies mentioned above, we did not find the TSD significant in the subset of T2 patients (*p* = 0.082). This may be due to the limited sample size. Further, the studies used USS imaging, which is highly operator dependent, whilst our study used MRI, a more objective form of imaging. TSD statistically significantly differed between node-positive and negative cancers in T3 patients (*p* = 0.0171).

Due to the breast screening programme in the UK, a significant proportion of cancers are detected at an earlier stage. It is worth exploring TSD in T1 and T2 tumours with a greater sample size in a prospective study. In our study, in 11 SLNB-positive patients (negative on diagnostic USS), 1 was stage T1 and 5 were T2. These tumours were all less than 17 mm from the skin.

There was a statistically significant negative correlation (*p* = 0.026) between tumour size and TSD in the node-positive group (i.e. larger tumours found closer to the skin) but no such correlation in the node-negative group. This suggests that the larger the tumour-breast volume ratio, the greater the likelihood of metastasis. However, due to the retrospective nature of this study, we did not have the breast size data to correlate the tumour-to-breast ratio with TSD.

A positive ER status was significantly associated with a positive nodal status (*p* = 0.007). Some studies have found no link between hormone receptor status and nodal positivity [17–19]. However, others [20, 21] found ER-positive breast tumours more likely to be node-positive. This may appear contradictory given the less aggressive nature of ER-positive breast cancer. However, the literature has reported that more aggressive tumours like triple-negative breast cancers have a greater propensity towards haematogenous spread [22, 23]

Our data also showed positive PR status to be significantly associated with positive nodal biopsy on univariate analysis, as demonstrated by Ureyen et al. [24]. However, this finding is inconsistent across all studies with positive PR status. PR status was also a missing variable amongst several patients due to the recent introduction of routine reporting of PR alongside ER and HER2 status; hence, it was not included in the multivariate analysis in this study.

Decisions for NACT used to be based on the Predict tool. Since then, the practice has moved away from NACT for ER + HER2- based on emerging data, including within our dataset. We selected 50 node-positives and 50 node-negatives regardless of receptor profile. More node-positives were seen in the (unselected) ER + HER2- cohort on analysis. Genomic testing data in node-positives was not available during the study period. In the adjuvant setting, we perform genomic testing in those borderline for chemotherapy based on clinical parameters.

Positive LVI had a significant association with nodal metastasis on univariate but not on multivariate analysis in our dataset. Moreover, TSD did not show any relationship to LVI status in the node-positive, node-negative, or whole cohort of patients. This questions the theory that tumours close to the skin more easily invade the lymphatic tissue, which is denser in the breast parenchyma closer to the skin. However, only 18 out of 95 breast tumours in our dataset were positive for LVI on histopathological analysis, which may be insufficient to show statistically significant relationships.

Greater tumour size has been associated with an increased risk of metastasis, though the relationship may not be linear [4]. The proliferative marker Ki-67 has also been associated with positive nodal status [17–19]. An increased rate of proliferation resulting in a larger tumour may increase the chances of tumour cells infiltrating surrounding lymphatics and metastasising to lymph nodes.

The tumour microenvironment also has a critical role in the behaviour and progression of cancers. Loi et al. conducted a prospective-retrospective study on triple-negative breast cancers. They found that higher levels of tumour-infiltrating lymphocytes at diagnosis correlated with decreased distant recurrence rates [25]. Further immune profiling studies have shown that in node-positive breast cancers, the immune profile is distinct from the node-negative breast cancers, whereby there is a shift towards T regulatory cells compared to CD8 + T cells and arrest of dendritic cell maturation [26–28]. Mahata et al. identified increased de novo steroid synthesis in T cells in vivo mouse models with tumour cell implantation. When T cell steroidogenesis was ablated in these models, both the primary tumour growth rates and metastatic colonisation decreased [7]. This production of steroids can increase the number and potency of Treg cells, highlighting the role of tumour cells on the immune environment and, in turn, metastatic potential. Others have identified differences in the sentinel node immune microenvironment of node-positive and -negative breast cancers, suggesting a role for early metastatic tumour cells in creating a pre-metastatic niche required for node metastasis [6, 29].

Pre-operative identification of those more likely to be node-positive may reduce the need to return to the theatre for axillary clearance. Avoiding second surgery will reduce adjuvant therapy delay and free up system capacity. Additionally, for those who are accurately identified to be node-positive and go on to the NACT pathway, knowledge of positive status would allow the axilla to act as an additional site for assessing response to NACT. It will also enable marking those identified nodes, leading to axillary conservation, such as targeted dissection, thus reducing axillary clearance rates.

## Conclusion

Our study shows that non-locally advanced tumours closer to the skin are associated with a greater risk of metastasis to the sentinel node. However, this measurement cannot be used in isolation to predict axillary lymph node metastasis. A significant correlation between ER-positive tumours (better prognostic biology) and nodal positivity appears contradictory. It suggests that there are factors contributing to nodal metastasis other than anatomical proximity and direct permeation into lymphatics. Given the borderline significance of TSD in our patient cohort, further exploration into TSD and other factors, including intra-tumoural microenvironments, such as immune cell-mediated steroidogenesis and inter-relation with immune-rich-nodal tissue, is required. To further test the significance of this relationship in T1 and T2 tumours, a prospective study with a larger cohort of T1/2 tumours would be beneficial. Moreover, as one of the very few studies to be carried out looking at TSD on breast cancer patients, we must follow this study up with a larger cohort patients in a prospective study. Additionally, pre-operative identification of those more likely to be node positive may suggest a second-look USS since a higher node stage may change therapeutic strategies such as upfront systemic therapy, node marking, and upfront ANC without returning to the theatre following SLNB.

## Data Availability

Enquiries about data availability should be directed to the authors.
